# Snakes, Snake Venoms, and Antivenenes1Presented before the American Veterinary Medical Association.

**Published:** 1902-06

**Authors:** E. M. Ranck

**Affiliations:** Philadelphia, Pa.


### SNAKES, SNAKE VENOMS, AND ANTIVENENES.[1]

By E. M. Ranck, V.M.D.,
PHILADELPHIA, PA.

The number of known species of snakes has been given as 1500 by some authorities, and as 1800 by others. The limit of their distribution seems to be the seventieth parallel, north latitude in

[1] Presented before the American Veterinary Medical Association.

Europe; the fifty-fourth in British Columbia, and the fortieth parallel, south latitude in the southern hemisphere. The number of species seems to be small in temperate zones, but increases toward the tropics, in which zones they are very abundant. Generally speaking, they may be, according to their habits, designated as: first, "burrowing snakes;" second, ground snakes; third, tree snakes; fourth, fresh water snakes; and fifth, sea snakes. The majority of snakes are active during the day, their energy increasing with the increasing temperature of the air; while some delight in the moist, sweltering heat of the tropical vegetation; others expose themselves to the fiercest rays of the mid-day sun. Those which inhabit temperate latitudes hibernate.

Snakes are the most stationary of all vertebrates. As long as a locality affords them a sufficiency of food and some shelter, to which they can readily retreat, they have no inducement to change it. Their organs of locomotion are the ribs, the number of which is very great, nearly corresponding to that of the vertebræ of the trunk. The locomotion is accomplished in the following manner: When a part of the body has found some projection of the ground which affords it a support, the ribs are drawn more closely together on alternate sides, thereby producing alternate bends of the body. The hinder portion of the body being drawn after, some part of it finds another support on the rough ground or projection, and the anterior bends being stretched in a straight line the front part of the body is propelled in consequence. The numerous ventral shields also add to this phenomena, and one ventral shield corresponds to one pair of ribs. They strike in much the same way, *i. e.*, by a dart of the anterior portion of the body which had been retracted in several bends. Snakes are oviparous and ovoviviparous; they deposit from ten to eighty eggs of an ellipsoid shape, covered with a soft, leathery shell in places where they are exposed to and hatched by moist heat. The parents pay no further attention to them, except the python, which incubate their eggs by coiling their body over them. In some families, as many fresh water snakes, the sea snakes, the vipers and crotalids, the eggs are retained in the oviduct until the embryo is fully developed. These snakes bring forth living young and are called ovoviviparous.

Since the snakes of North America interest us most, and especially the poisonous species, I will speak only of these. They comprise the crotalids, or "pit" vipers, which include the rattlesnake, copperhead, water moccason, and the elaps or coral snakes. All other snakes which abound in this country are non-poisonous.

The elaps or harlequin is a beautiful snake, having rings of red, black and yellow, and is so rare that I will omit this species in describing the details of snakes. Since we have so many " pit " vipers in this country, and in fact nearly all fatalities from snake bites can be traced to these, I will go into the details of some of the characteristics of this species.

The agkistrodon contortrix or copperhead is well-known to you all, and since it is so closely related anatomically to the rattler, I will pass this by, saying that since it is as a rule such a small snake, the bite is not generally fatal, owing to the fact that it secretes small amounts of venom. The agkistrodon piscivorus or water moccason, which is also related closely to the rattler, is larger and more dangerous and also deserves special mention, but the venom differs little from that of the rattlesnake, and so we will pass to the commonest family of the " pit " vipers, namely, the rattlesnake.

Perhaps a few of you may not know the meaning of the " pit," and this organ has been the topic of much discussion to many zoölogists. I will refer to what an authority on the subject says in reference to this peculiar organ. I refer to Lenord Stejnegar,[1] who says: " The name refers to the deep pit or hole found in the rattlesnakes, copperheads and water moccasons on the side of the face between the nostril and eyes. This cavity sinks deep into the maxillary bone and represents a " blind " sac lined with epidermis, and is not connected with any of the other cavities or organs in the head by any inside opening or canal. The external layer of the lining Leydig found to be a continuation of the outer skin, which, however, upon entering the cavity becomes thin and considerably modified. The surface is found to be composed of large, angular epidermis plates containing nuclei. The only conclusion that we can come to is that this organ is one of special sense. Wherein this " sixth " sense consists we do not know, nor do we know of anything in the habits of these snakes which would indicate its nature, or to what use the animal puts the organ."

The " pit " at once indicates a poisonous snake, and is characteristic of all our poisonous species of North America except the elaps. Another unfailing character is, of course, the presence of long, curved fangs in the anterior portion in the upper jaw. The fangs seem, on superficial examination, to lie in position resembling a knife blade one-fourth open, which must not be mistaken for being loose, for the fangs themselves are not movable; on the contrary, the viper's fangs are as solidly fixed in their sockets as are those of the

[1] Lenord Stejnegar: The Poisonous Snakes of North America.

elaps, but while in the latter the maxillary bones, into which the fangs are fastened, are elongated and horizontal, as in the harmless snakes, in the crotalids they are extremely shortened and appear to be in a vertical position.

The fang itself is a large, very pointed, and curved tooth, containing two cavities, the pulp cavity and the poison canal; the former situated on the concave side, the latter on the convex side of the tooth. The poison canal has a more or less slit-shaped opening near the base, on the anterior side of the fang, and another slit, narrower and longer, on the same side some little distance from the very sharply-pointed tip. Between these openings it is often possible to trace a more or less well-defined depressed line. A microscopic inspection of cross sections of the fang reveals the fact that the canal is nothing but a deep groove, the walls of which have closed over it anteriorly, the depressed line indicating the meeting of the wall or the seam. As a consequence of this origin of the canal, it is lined with the same hard layer of dentine as the outside of the fang, for it will be seen that this inner lining of the canal is in reality the anterior surface, while the outer layer is only the posterior surface of the normal tooth.

In the mouth, within the mucous membrane, and upon the pterygoid bone, lie, one behind and another below, the reserve fangs, each smaller than the one in front and less developed, until the situation of the last which is visible is marked by a minute papilla alone. There have been found as many as ten of these on each side of the mouth. When the fang is lost by natural process it is replaced within a few days; when violently displaced, several weeks sometimes elapse before the next fang is fixed firmly enough to be useful to the snake. If the functional fang be lost or shed, the next tooth gradually assumes its position, and finally occupying the place of the lost one, becomes ankylosed.

There are several muscles used in erecting the fang and also the depression of same. The elevator muscle of the fang is the sphenopterygoid muscle which arises along the median ridge of the base of the skull, and running backward is inserted upon the enlarged posterior end of the pterygoid bone. The contraction of this muscle pulls the pterygoids forward, which thus push the lower end of the maxillary forward, the upper end being held in position by the lachrymal hinge. The tips of the fang, describing part of a circle, finally point downward instead of backward. The chief retractor muscle, which antagonizes the elevator muscle by acting in the opposite direction, is the external pterygoid muscle, which, arising from the joint between the quadrate bone and the lower jaw, runs forward and is inserted on the outside of the maxillary bone a little below the joint of the latter with the outer pterygoid bone. It will be seen that contraction of this muscle means a pulling backward of the maxillary bone, resulting in the backward and upward movement of the point of the fang.

Close observation will disclose the fact that a snake when ready to strike its prey, *i. e.*, in coiled position, never has its mouth open, neither can it throw its venom, but when in captivity I have noticed it striking against glass, on which it deposits its venom. Another interesting feature of these snakes at least is the presence of an organ known as the "rattle." Unlike the tails of some snakes, gradually tapering to a point covered by a cone-shaped, more or less acute scale, this species continues rather thick to where the rattle is appended. The rattle consists of numerous rings of dermal, horny consistency, and at each shed of the skin simply retains some of its shed, which adds another button to its rattle. Since the rattle is such a fragile organ, one can easily see how erroneous the old method of determining the age of a snake by its rattle is, and you may find quite old snakes with very few rattles, they having been destroyed by blows, etc. A peculiar fact can be observed that when a snake is moving or crawling along it does so with its tail elevated, which, in a measure, protects the rattle from dragging. In captivity snakes have been known to rattle for hours continuously, and the sound is produced by a vigorous shaking and vibrating of the end of the tail, the dead, horny cones producing a noise resembling that of a locust. Dr. Foektistow states that in a vigorous rattle the tail makes seventy-five vibrations, and the rattle 110 vibrations per minute.

What is the object of the rattle? This question has never been satisfactorily solved; surely, it has caused the snake's extermination to a great extent, especially in cultivated districts. Some philosophers say that since it resembles the noise of the cicada rimosa, the snake used this to decoy insect-eating birds. Others say it is a warning to innocent animals and man. Professors Putnam and Shaler suggested the function to call sexes together. The charming powers of snakes need no mention here, since this erroneous supposition is only for the superstitious.

In the same manner as the gallow fang is developed from the grooved fang, and this again from the plain, solid tooth, so is the poison gland evolved from the ordinary salivary gland by a specialization of the yellow portion of the latter.

The typical venom gland is found in its fullest development among the pit vipers, and is located on each side of the head below and behind the eye. The shape is that of a flattened almond, the pointed end toward the front and below the eye, tapering to a narrow duct which carries the poison to the inlet at the base of the fang.

The interior structure of the gland may be described briefly as consisting of a basal cavity into which the small ducts of the glands open. These ducts run toward the walls of the gland, branching and finally ending in minute blind bags, the whole system of ducts being supported by a network of numerous fine threads and thin sheets of fibrous tissue. The ducts are lined with a more elongated epithelium, the blind pouches with angular epithelial cells. The external covering of the gland is made up of two more or less distinct layers of fibrous tissue, the outer one being continued posteriorly in a ribbon-like ligament, running backward and inserting itself upon the joint of the jaw. A short ligament on the side facing the skull attaches the gland firmly to the latter, and a third one below connects with the external pterygoid muscle. At the anterior end of the gland, the capsule, the base of which meets the upper opening of the canal, continues as the outer covering of the duct which carries the poison from the gland to the fang. The duct in its normal position makes a sudden upward curve under the eye, descending from which it follows the posterior wall of the pit and finally passes over the rounded outer front edge of maxillary bone, at the base of which it meets the upper opening, or inlet, of the canal through the fang.

The whole poison apparatus has been very appropriately compared to a hypodermic bulb syringe, the needle, with its obliquely-cut point and slit-like outlet, representing the fangs, the bulb corresponding to the poison glands, the muscles of the hand which presses the bulb performing the same task as the anterior temporal muscle.

Now, since all snakes which menace the lives of people and animals are provided with the terrible organs of offence and defence, it is naturally interesting to know something about the terrible product which is a secretion of part of the mechanism of these creatures. Many eminent scientists have from time to time experimented with these products, studying them in relation to the chemical constituents they contain, as well as the physiological action on lower animals.

In 1883 Drs. S. Weir Mitchell and E. T. Reichert, of Philadelphia, Pa., laid before the National Academy a preliminary report of the result of their studies, which after a lapse of twenty years

Dr. Mitchell has resumed. He imagined that owing to the complexity of the symptoms in snake poisoning, the cause probably was due to more than venom alone. He states in a popular report some of the following facts: 1. When a little of the venom is placed in sufficient water it dissolves readily. 2. Heating coagulates. 3. Heating attenuates the venom. 4. By dialysis he gets a venom peptone, and later a venom globulin. These experiments have been confirmed by Wolfenden, who found in the cobra a large quantity of the globulin, but very little albumose. Recently (1895), Frazer has experimented with the venom of the cobra of India and rattlesnakes of America, and has been able to immunize the animal against thirty times the minimum toxic quantity, and furthermore states that from these animals he found the antitoxin which he calls antivenene.

Calmette has contributed largely to our knowledge of the action of snake venom, of which he says. 1. Dilute solutions of hypochlorite of lime and of chloride of gold destroy or neutralize venom in vitro. 2. The toxicity of the venom is not destroyed by a temperature of 80°. 3. The blood of all snakes, of the salamander, and of the toad is poisonous. 4. The toxicity of the blood is destroyed by a temperature of 68°, therefore the toxicity of the blood and that of the venom is not due to the same constituent. 5. An animal may be immunized against the venom or against the blood, but immunity to the venom does not give immunity to the blood. 6. Possibly some immunity is possessed by the hog, but there was no opportunity to demonstrate this. 7. An animal immunized against venom is not immune to tetanus, while antitetanus serum from the horse does retard or wholly prevent the action of venom.[1]

The above-named scientist has contributed to the public an antivenene which does, without doubt, save an animal's life against certainly fatal doses of the venom.

In April, 1899, Professor Joseph McFarland and myself started an experiment for the H. K. Mulford Company on the same lines as Professor Calmette, using horses which were gradually immunized until the quantities of the venom they could withstand far exceeded that which would certainly have been fatal at initial injection. The following is a short clinical record which I have observed from time to time while conducting these experiments, which may interest some of you to whom cases are brought from time to time for diagnosis and treatment, which are suffering from snake bites.

The clinical symptoms attending the injection vary according to

[1] Vaughn and Novy: Ptomains and Leucomains.

the method of injection. If it be subcutaneous, the venom being very irritating, causes much inconvenience the moment the lymphatics start to take it up, and from this time on the animal suffers great pain. Soon after the injection inflammation takes place, with the attending phenomena, swelling, pain, etc. The termination of this swelling may be suppurative or by absorption, and this again varies in different horses or at different times in the same horse. One great peculiarity about these infiltrations is the large area affected; for instance, if a horse be injected on the left side above the superior border of the scapula the infiltration will often extend anteriorily to middle third of cervical region, and the entire arm and often forearm on the same side, posteriorly to middle of dorsal region from backbone to abdomen, and very often continue upward on side opposite injection.

The animal suffers during respiratory movements, so the latter are almost entirely abdominal and usually shallow. The animal rarely assumes decubitus position, since any excessive movement is attended with great pain. Suppuration takes place, the subcutaneous tissue and skin remain hard while the melting down process is progressing underneath, so that unless the abscess is deeply cut into the whole area will slough out. The temperature usually remains normal until pus forms, but animal often breaks out in violent perspiration which feels cold to the hand, while the infiltration is very hot and dry, the line of demarcation being sharply drawn and often raised one-half to one inch. Post-mortem shows tissues involved very black, resembling that of a bruised muscle with ecchymotic spots distributed through, and here, too, the line of demarcation is even more closely discernible. The formation of pus is usually attended by the production of gas which is very offensive and nauseating, the exact nature of the micro-organism causing this gas production I am not prepared to define. If pus is not formed by the end of three days after the injection the horse will gradually resume eating, although appetite is often fickle, and the infiltration will pass away in from five to seven days. The infiltration sometimes extends to the extremities, and when this is the case the articulations are largely involved; palpation shows entire joint enlarged, swollen, œdematous, and of a doughy consistency, pits on pressure, and usually very sharply outlined. This, however, passes away in about two weeks. When horses or cattle are bitten by rattlesnakes, the usual seat of injury being the extremities, they swell enormously and often result in a chronic cellulitis or elephantiasis from which they never recover. This chronic condition is noticed also in horses when they are injected frequently with small doses of the venom in the same area, even if on side of neck or shoulder, but it is not so noticeable as if it were on extremities.

When the animal is injected intravenously the case is somewhat different than that from subcutaneous injections. I usually place him in slings preparatory to injecting, the seat of which may be any large subcutaneous vein; cleanse and prepare place of injection in usual way, and in every case use large dilutions, since small concentrated solutions are far more dangerous, especially when thrown into the circulation. This fact, by the way, explains why an animal is often killed almost instantly when bitten—*i. e.*, on account of reaching the circulation at once, or if it be a small animal, either the pleural or peritoneal cavities, hence rapid absorption and death. The dose varies according to the degree of immunity the horse has attained and the violence of the previous reaction, and not to the length of treatment, as we often have to decrease the dose and lengthen the intervals between injections, depending upon the condition of the individual animal. The reaction takes place as soon as the venom reaches the circulation, but not violent at first, since the dilutions are very great, and doses graded according to the animal under treatment, besides when giving large amounts of any liquid intravenously I prefer giving by gravity method and with a needle of fairly small calibre; in this way the progress of material is retarded and enters the circulation slowly, so by the time the last part of solution has entered the blood the first part has probably made the entire circuit.

The first reaction usually noticed from intravenous injection is a cough, often violent, then uneasiness, pawing, shaking head, and in general resembling symptoms shown by an animal which you have bled to his limit before fainting. After this disappears, and it lasts but a few moments, the horse makes a plunge forward (if not protected will injure himself, being at this stage entirely unconscious), and if allowed to go down on the floor will lie for a few seconds apparently quiet, then usually have a spasm. In one case I noticed one which suddenly rolled on his back with his four feet in the air, and, with muscles cramped, held them in this position for a few minutes, and in probably five minutes was on his feet. Following all these injections intravenously, there will be noticed defecation, the feces often covered with mucus of a bloody tinge, and the greater the amount of this elimination the shorter is the time of the attack. If one is not cautious when injecting intravenously, some of the venom solution will enter the subcutaneous tissues or jugular gutter, and then the swelling is very pronounced and often gives much trouble, so while the needle is in the jugular vein it is well to gently manipulate the tube through which the solution passes, in this way avoiding the trouble above mentioned.

The idea of those experiments is, of course, to produce an antitoxin for snake bites, and such has been accomplished by Calmette and others abroad, but as yet we have not succeeded in producing a product of as great strength as the above-named scientists. However, the plan outlined is practicable, and we have one horse which has yielded some fairly high-grade serum, *i. e.*, some which will, in small quantities, certainly protect an animal against fatal amounts of the venom.

The other antidotes which are commonly used are chloride of lime or gold, 10 per cent. solution, ten to twenty drops injected in and around the place bitten; these products neutralizing the venom so that it is rendered non-poisonous. The general treatment is strychnine pushed to the therapeutic limit; given in any way, but probably best hypodermically, since it is absorbed more readily. The actual cautery is efficient, but not practical on account of the scar and pain, and must be very deep to be of any use, especially when bitten by a large snake, since the fangs are inserted deep in the flesh. Ligatures should be placed about the member as soon as possible to prevent general absorption and circulation until the neutralizing remedies are secured and administered. Perhaps at some future time when science is called upon to solve these problems there will be some standard remedy for these terrible poisonings, and we hope in the near future to place before the profession some antivenene which will be as reliable as the now universally used diphtheria antitoxin.

